# GLP‐1 and GIP Beyond Glycaemia: Evolutionary Roles in Energy Optimisation and Organ Protection

**DOI:** 10.1002/dmrr.70108

**Published:** 2025-11-16

**Authors:** Dario Tuccinardi, Mikiko Watanabe, Davide Masi, Daniele Gianfrilli, Silvia Manfrini, Carel W. le Roux

**Affiliations:** ^1^ Fondazione Policlinico Universitario Campus Bio‐Medico Rome Italy; ^2^ Research Unit of Endocrinology and Diabetology Department of Medicine and Surgery Università Campus Bio‐Medico di Roma Rome Italy; ^3^ Department of Experimental Medicine Section of Medical Pathophysiology Food Science and Endocrinology Sapienza University of Rome Rome Italy; ^4^ Diabetes Complications Research Centre Conway Institute University College Dublin Dublin Ireland

**Keywords:** cardiometabolism, diabetes, GIP receptor, GLP‐1 receptor, incretin, obesity

## 1 Introduction

Incretin hormones, primarily glucagon‐like peptide‐1 (GLP‐1) and glucose‐dependent insulinotropic polypeptide (GIP), have traditionally been viewed within the endocrine paradigm of nutrient‐stimulated insulin secretion. Yet, their receptors, GLP‐1R and GIPR, in organs far beyond the pancreatic islets raise a broader question: why are these intestinal hormones sensed by cardiomyocytes, endothelial cells, renal tubular structures, and even immune cells? The answer, we propose, lies in a dual physiological and evolutionary rationale that extends the role of these hormones from metabolic effectors to systemic sentinels, safeguarding vital organs from postprandial metabolic and vascular stress.

Recent discoveries in incretin biology, both from preclinical models and outcome trials, support a paradigm in which GLP‐1 and GIP coordinate two interlinked but conceptually distinct biological mandates: the optimisation of postprandial energy handling [1], and possibly the protection of vital organs from the transient stress of nutrient ingestion [2]. Although separable for analytical purposes, these functions likely evolved in parallel to maximise metabolic efficiency and minimise nutrient‐induced tissue stress.

## 2 Postprandial Energy Optimisation

GLP‐1 and GIP are secreted from enteroendocrine L‐ and K‐cells, respectively, within minutes of nutrient exposure in the small intestine. This rapid release underscores their function as anticipatory signals acting before nutrient absorption is complete. Traditionally, these hormones enhance insulin secretion in a glucose‐dependent manner and suppress glucagon release, thereby improving postprandial glucose clearance while reducing the risk of hypoglycaemia [1]. However, their actions extend beyond pancreatic islets. GIPR is highly expressed in adipose tissue and facilitates lipid storage during energy excess by increasing lipoprotein lipase activity and promoting adipocyte insulin sensitivity [3]. By contrast, GLP‐1 slows gastric emptying and reduces appetite via central and vagal pathways [4], contributing to energy intake and storage coordination.

Crucially, incretin signalling seems to regulate the secretion of metabolic hormones and the distribution of blood flow to specific tissues during the postprandial period. GLP‐1 enhances microvascular perfusion in skeletal muscle, improving glucose uptake and insulin sensitivity [5]. GIP enhances blood flow to adipose tissue, promoting triglyceride deposition and local nutrient assimilation [3, 6]. These vasodilatory effects are physiologically directed; they form a precisely regulated system to supply organs based on their immediate energy or functional needs: skeletal muscle and myocardium receive increased perfusion for contractile activity, adipose tissue for lipid storage, and the kidney for filtration and clearance. The approval of tirzepatide, a dual GIP and GLP‐1 receptor agonist, has demonstrated the clinical potential of targeting this dual‐incretin axis. Tirzepatide not only reduces HbA1c and body weight more effectively than selective GLP‐1R agonists but also improves insulin sensitivity and lipid metabolism, suggesting that GIP may enhance the energy‐storing capacity of peripheral tissues in synergy with GLP‐1's regulation of intake and insulin dynamics [7].

**FIGURE 1:**
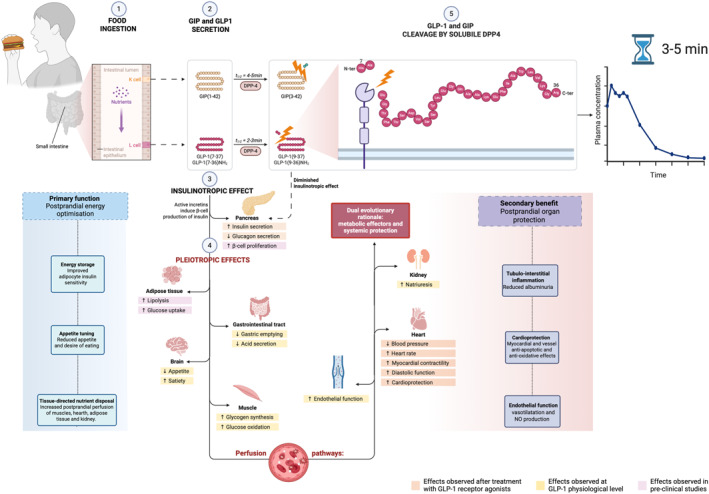
Dual physiological role of GIP and GLP‐1 as postprandial metabolic effectors and systemic protectors. Incretin hormones are secreted within minutes of nutrient ingestion and act as short‐lived, meal‐coupled signals. The schematic highlights their two complementary roles: (i) postprandial energy optimisation, coordinating insulin secretion, nutrient storage, satiety, and tissue‐specific perfusion; and (ii) postprandial organ protection, mitigating haemodynamic, vascular, and inflammatory stress through renal, cardiovascular, and endothelial actions.

These insights support the hypothesis that incretin hormones regulate glucose and orchestrate an organ‐specific vascular and metabolic response to feeding, a physiological mechanism evolved to optimise energy delivery and storage across key tissues (Figure 1).

## 3 Postprandial Organ Protection

While efficient nutrient absorption is essential, excessive nutrient intake presents notable challenges. High‐fat and high‐sugar meals are linked to temporary increases in circulating lipids, glucose, and endotoxins, which can trigger systemic oxidative stress, endothelial dysfunction, and inflammatory activation [8, 9].

Importantly, even in ancestral settings lacking chronic caloric excess, nutrient intake itself likely acted as a physiological stressor. The postprandial state naturally involves transient glycaemic variability, oxidative stress, and free fatty acid (FFA) fluctuations, which can disrupt endothelial homoeostasis. Therefore, although essential, eating requires immediate compensatory mechanisms to preserve vascular and organ integrity.

GLP‐1 and GIP act as protective signals in this setting. GLP‐1 R is expressed in vascular endothelial cells, where its activation increases nitric oxide production and promotes vasodilation [10, 11]. These effects have been shown in humans, where GLP‐1 infusions enhance microvascular blood flow [11, 12, 13].

Similarly, GIPR is expressed in atherosclerotic plaques and appears to modulate macrophage activation [14, 15] and vascular inflammation [16].

Cardiomyocytes and renal tubular cells also express incretin receptors. In models of myocardial ischaemia‐reperfusion, GLP‐1R agonists reduce infarct size, inhibit apoptosis, and attenuate oxidative injury [17]. In the kidney, GLP‐1R activation has been shown in preclinical models to reduce albuminuria, improve glomerular haemodynamics, and suppress tubulointerstitial inflammation, with supportive evidence also emerging from human trials and mechanistic studies in cultured human renal cells [18].

GLP‐1 and GIP coordinate the efficient postprandial allocation of nutrients to metabolically active tissues while simultaneously mitigating vascular and inflammatory stress. This dual action reflects an evolutionary strategy to optimise nutrient use while protecting tissues after meals. Their very short half‐life (1–2 min), due to DPP‐4 degradation, confines their action to the immediate post‐meal period, consistent with their role as transient signals for energy coordination and organ protection. Originally, their effects—such as increasing perfusion to muscle, heart, and kidney—served to meet postprandial energy demands in ancestral settings. Today, in contexts of chronic overnutrition, these same mechanisms help protect organs from metabolic damage, particularly in cardiometabolic disease. Therefore, what evolved to ensure energetic efficiency now provides a physiological buffer against cardiovascular and renal stress (Figure 1).

## 4 Origins and Evolution of Incretin Signalling: From Embryonic Crosstalk to Survival Signals

The widespread distribution of incretin receptors across various organ systems suggests a profound evolutionary and developmental rationale. From embryogenesis to survival in fluctuating nutrient environments, the GLP‐1 and GIP systems reflect conserved strategies for energy sensing and organ function protection.

The presence of GLP‐1R and GIPR in non‐metabolic tissues may originate from early developmental crosstalk between the endoderm and mesoderm. While the gut, pancreas, and liver derive from the foregut endoderm, the heart, kidney, vasculature, and immune system are from the mesoderm. During organogenesis, mesodermal structures such as endothelium and cardiac mesenchyme influence endodermal progenitors, inducing key transcription factors like PDX1 and PTF1A, which are essential for pancreatic development. These reciprocal interactions may have established shared receptor‐ligand systems, including incretin signalling, which persist into adulthood and are repurposed for systemic nutrient sensing and organ protection.

The evolutionary dimension of this system is equally compelling. The genes encoding GLP‐1 and GIP derive from ancient precursors with highly conserved exon–intron structures that existed before the divergence of vertebrates (single‐copy genes with conserved flanking regions across mammals and vertebrates) [19, 20]. GLP‐1 and GIP receptors are present in the brains of rodents and primates, particularly in hypothalamic and brainstem nuclei that regulate feeding and autonomic control, indicating an ancestral neuroendocrine function [19].

In invertebrates such as Drosophila melanogaster, short neuropeptide F (sNPF), a peptide structurally related to vertebrate neuropeptide Y (gut‐derived anorexigenic hormone), plays a key role in regulating food intake and body size. Gain‐ and loss‐of‐function models demonstrate that sNPF acts in the nervous system to modulate feeding behaviour and growth parameters. This suggests a conserved role for neuropeptide signalling in energy balance regulation [21]. A striking example of evolutionary convergence is the Gila monster (*Heloderma suspectum*), whose salivary peptide exendin‐4 binds mammalian GLP‐1R with high affinity. Secreted in response to infrequent large meals, this peptide enhances nutrient handling and inspired the development of GLP‐1 receptor agonists such as exenatide [22].

**FIGURE 2:**
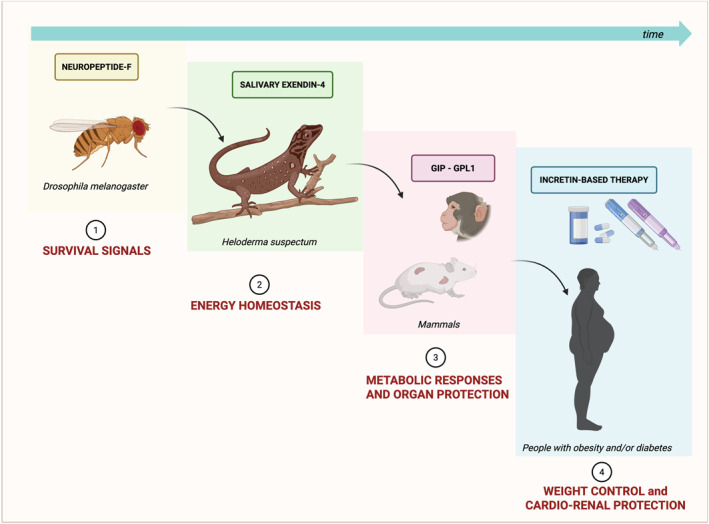
Evolutionary and developmental origins of incretin signalling as a dual system for metabolic coordination and organ protection. Incretin hormones have evolved from ancient neuropeptidergic systems regulating energy balance in primitive organisms (e.g., Neuropeptide F in *Drosophila*) to complex endocrine signals in vertebrates (GLP‐1, GIP) that couple nutrient sensing with systemic homoeostasis. Their genes are derived from highly conserved ancestral sequences predating vertebrate divergence. During vertebrate organogenesis, shared molecular pathways between endodermal (gut) and mesodermal (heart, kidney, vasculature) lineages enabled broad receptor expression across metabolic and non‐metabolic tissues. In higher organisms, GLP‐1 and GIP coordinate postprandial responses not only via insulinotropic and anorexigenic effects but also through direct actions on cardiomyocytes, endothelial cells, and renal structures, conferring vasodilation, natriuresis, and anti‐inflammatory effects. This trajectory—from embryonic crosstalk to evolutionary pressure—has culminated in a hormone system with both anticipatory and protective roles, now harnessed in pharmacotherapy for diabetes, obesity, and cardiorenal diseases.

These developmental and evolutionary insights suggest that incretin signalling evolved as a rapid, meal‐coupled adaptation, originating from embryonic gene networks and shaped by selective pressures to balance nutrient absorption with systemic protection. In modern contexts of caloric abundance, the same pathways now serve as therapeutic targets to buffer cardiometabolic and renal stress. This unifying framework, rooted in both ontogeny and phylogeny, underlines the extraordinary versatility and relevance of the incretin axis in human physiology and disease (Figure 2).

## 5 Clinical Implications

The dual‐function model of incretin action has direct implications for drug development. GLP‐1 receptor agonists are already established in managing type 2 diabetes and obesity, with a growing evidence base in cardiovascular and renal protection. Trials such as LEADER (liraglutide), SUSTAIN‐6 (semaglutide), and REWIND (dulaglutide) consistently report reductions in major adverse cardiovascular events (MACE), hospitalisation for heart failure, and progression of kidney disease, often before significant weight loss or glycaemic improvements are achieved [16, 17, 18].

These findings challenge the traditional view that metabolic improvements mediate cardiovascular benefits and instead support the idea of direct effects on organs. Further evidence for an extraglycaemic effect of GLP‐1 receptor signalling comes from the FLOW trial (*n* ≈ 3500), where semaglutide significantly slowed the progression of kidney disease in patients with type 2 diabetes and high renal risk, reinforcing the hypothesis of a direct renoprotective action [19]. Even more striking is the recent SELECT trial (*n* ≈ 17,600), which demonstrated that semaglutide reduced MACE in individuals with obesity but without diabetes, confirming that the cardiovascular benefit is not merely a function of improved glucose control [20].

Tirzepatide's ability to amplify metabolic and non‐metabolic benefits further supports the dual‐rationale model. In SURPASS‐4, tirzepatide reduced albuminuria and improved eGFR independently of glycaemic control, indicating renal effects that may derive from its vascular or anti‐inflammatory actions [23].

Understanding this biology also opens new paths for precision medicine. Genetic variants in the GLP1R locus have been associated with differential glycaemic and weight responses to GLP‐1RAs [24, 25], and ongoing studies are examining whether similar variation modulates cardiovascular outcomes.

## 6 Conclusion

Incretin hormones do not merely facilitate glucose homoeostasis; they coordinate an integrated systemic response to feeding that aligns metabolic efficiency with the protection of organs. The evolutionary rationale for this dual purpose is compelling: in environments of fluctuating nutrient availability, the ability to safely store energy without causing vascular or renal damage would have conferred a survival advantage.

The co‐evolution of endocrine signalling and organogenesis gives rise to a class of hormones uniquely adapted to detect nutrients and protect against their collateral damage. GLP‐1 and GIP therefore emerge not only as incretins, but as systemic sentinels and anticipatory agents that sustain homoeostasis across multiple physiological axes.

As our understanding deepens, our capacity to harness these hormones for multidimensional therapies will also grow, customised to glycaemic control and the integrated protection of the heart, kidney, and vasculature.

## Author Contributions

D.T. conceptualized the manuscript and drafted the initial outline. D.T. and M.W. wrote the article. D.M., D.G. and S.M. contributed to the literature review, figure design, and critical revisions of the text. C.W.L.R. revised the manuscript for intellectual content. All authors reviewed and approved the final version of the manuscript.

## Funding

This study was supported by European Union through Italian Ministry of University and Research (PRIN2022NS7PRM).

## Conflicts of Interest

The authors declare no conflicts of interest.

## Peer Review

The peer review history for this article is available at https://www.webofscience.com/api/gateway/wos/peer-review/10.1002/dmrr.70108.

## Data Availability

Data sharing not applicable to this article as no datasets were generated or analysed during the current study.
